# Tislelizumab combined with Anti-GD2 antibody-based chemoimmunotherapy for pediatric relapsed/refractory high-risk neuroblastoma: a two-Case Report

**DOI:** 10.3389/fphar.2026.1890595

**Published:** 2026-08-05

**Authors:** Ting Zhang, Xuelian Liao, Yanhua Li, Jingwei Yang, Jingbo Shao, Huanhuan Zhang, Shayi Jiang

**Affiliations:** 1 Department of Hematology and Oncology, Shanghai Children’s Hospital, School of Medicine, Shanghai Jiao Tong University, Shanghai, China; 2 Department of Radiology, Shanghai Children’s Hospital, School of Medicine, Shanghai Jiao Tong University, Shanghai, China

**Keywords:** chemoimmunotherapy, neuroblastoma, pediatric, refractory, relapse, tislelizumab

## Abstract

Neuroblastoma (NB) is one of the most common extracranial solid tumors in children. Patients with high-risk metastatic disease remain at substantial risk of relapse and have poor long-term outcomes. No standard salvage regimen has been established for relapsed/refractory (R/R) NB, and the role of immune checkpoint inhibitors in pediatric NB remains investigational. Here, we report two pediatric patients with R/R high-risk NB who received a tislelizumab-containing multi-agent regimen consisting of tislelizumab, an anti-GD2 monoclonal antibody, GM-CSF support, and sequential mICE/VIT chemotherapy. The anti-GD2 antibody, dinutuximab beta or naxitamab, was selected according to each patient’s insurance coverage. Both patients tolerated treatment well, and no severe immune-related adverse events were observed during the reported follow-up period. After four cycles of combination therapy, Patient 1 showed marked regression of soft-tissue lesions and clearance of bone marrow involvement. Patient 2 achieved a complete response and remained disease-free during the 5-month follow-up period after treatment completion. However, the durability of this response remains uncertain because of the limited follow-up duration. These preliminary short-term observations suggest that this multi-agent regimen may be a feasible salvage approach for selected pediatric patients with R/R high-risk NB. However, the durability of these antitumor responses cannot be confirmed given the limited surveillance period. Notably, this retrospective two-case observation cannot distinguish the independent antitumor contribution of tislelizumab alone, as multiple therapeutic components were administered simultaneously without control groups to isolate single-agent efficacy. Larger prospective studies are warranted to further evaluate the efficacy and safety of this approach.

## Introduction

Neuroblastoma (NB) is an extracranial solid tumor arising from the developing sympathetic nervous system. The incidence rate is 7.72 per million children aged 0–14 years (Ni et al., 2022), and NB accounts for 12%–15% of childhood cancer deaths (Gatta et al., 2014; Nong et al., 2025). Despite multimodal therapy including chemotherapy, surgery, radiotherapy, and autologous stem cell transplantation, the 5-year event-free survival (EFS) rate for high-risk NB remains suboptimal, at 37.7% (Pinto et al., 2015; Su et al., 2020), and patients with relapsed or refractory disease have an extremely poor prognosis.

Disialoganglioside GD2 is a cell-surface glycolipid. It is highly expressed on most NB cells, with limited expression in normal tissues (Machy et al., 2023), and is an established target for immunotherapy in patients with NB. Anti-GD2 antibodies have become standard therapy for high-risk NB (Bagatell et al., 2024), and their combination with chemotherapy improves response rates in R/R disease, with an overall objective response rate of 45% and prolonged event-free survival (Olgun et al., 2025). Nevertheless, further optimization of salvage strategies is needed for patients who progress after anti-GD2 antibody-based therapy. In mainland China, dinutuximab beta (approved 2021) and naxitamab (approved 2022) are available for indicated pediatric patients with high-risk or R/R NB.

Immune checkpoint inhibitors targeting programmed cell death protein 1 (PD-1) have become established treatments for several adult malignancies and are increasingly being explored in pediatric solid tumors (Que et al., 2023). Although basal PD-L1 expression is generally low in NB cells, it may be induced under certain therapeutic or inflammatory conditions. Preclinical studies demonstrate that anti-GD2 antibodies upregulate PD-L1 on NB cells, and that the combination of PD-1 blockade and anti-GD2 antibodies may produce synergistic antitumor effects, providing a strong rationale for combined immunotherapy (Siebert et al., 2017). Published clinical experience with PD-1 inhibitors combined with anti-GD2-based immunotherapy in pediatric R/R high-risk NB remains very limited, with only small retrospective case series and early phase preliminary data reported to date (Zsigrai et al., 2025).

In this study, we administered tislelizumab (Xu et al., 2023) combined with anti-GD2 antibody-based chemoimmunotherapy (a multi-agent regimen consisting of tislelizumab, an anti-GD2 monoclonal antibody with GM-CSF support, and alternating mICE/VIT chemotherapy; dinutuximab beta or naxitamab was selected according to each patient’s insurance coverage) to two pediatric patients with R/R high-risk NB who had experienced multiple relapses and failed multiple prior lines of therapy. We describe their clinical responses and treatment tolerability, providing preliminary clinical observations to support future investigation of this strategy.

## Case description

### Patient 1

**FIGURE 1:**
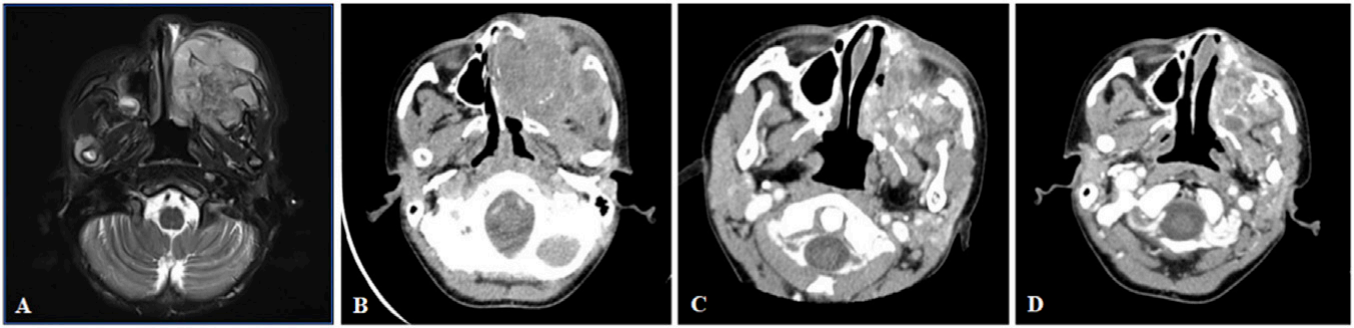
Imaging findings of Patient 1. **(A)** Maxillofacial MRI acquired in May 2025 shows local tumor recurrence. **(B)** Contrast-enhanced CT performed in July 2025 after two cycles of salvage ICE chemotherapy showed progressive maxillofacial soft-tissue disease; the lesion measured 63.5 mm × 55.6 mm × 65.7 mm. **(C)** Follow-up contrast-enhanced CT in October 2025 after two cycles of tislelizumab-containing chemoimmunotherapy showed marked tumor regression, with the lesion reduced to 41.4 mm × 44.6 mm × 41.6 mm. **(D)** Contrast-enhanced CT in January 2026 after four cycles of combination therapy; the lesion measured 40.6 mm × 30.7 mm × 38.8 mm.

In March 2018, a 22-month-old boy presented with recurrent fever and was found to have a posterior mediastinal mass. Bone marrow cytology and flow cytometry confirmed metastatic NB with MYCN amplification, and the disease was classified as stage M according to the International Neuroblastoma Risk Group Staging System (INRGSS). The patient received four cycles of induction chemotherapy according to the SCMC-NB-2016 protocol, followed by complete surgical resection of the mediastinal mass and six subsequent cycles of adjuvant chemotherapy using the same regimen (the SCMC-NB-2016 protocol, an institutional risk-stratified regimen using alternating six-agent chemotherapy combinations, as detailed in Table 1). After 6 months of isotretinoin maintenance therapy, the patient achieved first complete remission (CR). Five years after first CR (April 2024), recurrent fever emerged, and bone marrow evaluation confirmed tumor relapse. PET/CT revealed multifocal disease, including spinal lesions adjacent to the right posterior third to fifth ribs, a left maxillary sinus mass, hypermetabolic deep cervical, submandibular and parotid lymph nodes, and extensive systemic bone metastases. The patient received five cycles of dinutuximab beta combined with the irinotecan-temozolomide (IT) regimen. Subsequent MIBG scintigraphy showed focal tracer uptake in the skull and right maxillary sinus, as well as suspicious vertebral uptake, and a MIBG Curie score of 6, followed by local radiotherapy targeting the left temporal bone and scapula. After an initial response, MRI performed in May 2025 confirmed progressive disease (PD) (Figure 1A). Further bone marrow evaluation showed tumor cells comprising 47.5% on cytology, with CD56^+^/CD81^+^/GD2^+^ cells accounting for 15.5% by flow cytometry, and bone marrow biopsy confirmed bone marrow involvement by NB. The patient was admitted to our hospital for further treatment and received two cycles of salvage ifosfamide, carboplatin, and etoposide (ICE) chemotherapy. However, the facial mass continued to enlarge after two cycles of salvage ICE chemotherapy (Figure 1B). Two months after disease progression (July 2025), tumor biopsy confirmed GD2 positivity. Immunohistochemical PD-L1 testing was performed on this biopsy specimen, showing a tumor proportion score (TPS) < 1% and a combined positive score (CPS) of 5, providing limited baseline biomarker information before subsequent combined chemoimmunotherapy (Table 1).

**TABLE 1:** Clinical characteristics and treatment courses of the two patients.

Calendar period	Relative timing	Disease status/response	Major treatment or assessment
Patient 1: 9-year-old boy at initiation of tislelizumab-containing therapy			
Mar 2018–March 2024	0–72 months after initial diagnosis; first CR maintained for approximately 60 months	► Posterior mediastinal NB, INRGSS stage M, MYCN-amplified ► First CR	► SCMC-NB-2016 protocol ► Tumor resection in June 2018 ► Isotretinoin maintenance for 6 months
Apr 2024–April 2025	First relapse occurred 5 years after first CR; 0–12 months after first relapse	► First relapse ► PR followed by PD	► Dinutuximab beta plus irinotecan/temozolomide ► Local radiotherapy to the left temporal bone and scapular region
May–June 2025	Approximately 13 months after first relapse; 3 months before initiation of tislelizumab-containing therapy	► PD	► Maxillofacial soft-tissue tumor biopsy ► ICE chemotherapy
Aug 2025–January 2026	0–5 months of tislelizumab-containing combination therapy; initiated 3 months after PD confirmation	► PR; bone marrow CR by cytology and flow cytometry	► Tislelizumab, naxitamab, GM-CSF, and sequential mICE/VIT chemotherapy
Feb 2026 onward	After completion of four cycles of combination therapy	► Persistent PR	► Concurrent chemoradiotherapy
Patient 2: 14-year-old girl at initiation of tislelizumab-containing therapy			
May 2020–June 2022	0–26 months after initial diagnosis	► Retroperitoneal NB, INRGSS stage M, MYCN non-amplified ► First CR	► Chemotherapy ► Tumor resection ► Local radiotherapy ► Allo-HSCT ► Dinutuximab beta-based immunotherapy
Aug 2023–September 2024	First relapse occurred 38 months after initial diagnosis and 14 months after first CR	► First local relapse ► Second CR	► Two tumor resections ► Chemotherapy ► Local radiotherapy (intracranial SBRT in September 2023; mediastinal/pleural VMAT in January 2024) ► Dinutuximab beta-based immunotherapy
Dec 2024–May 2025	8 months after second CR	► CR	► Oral eflornithine
Jun–December 2025	Second relapse occurred 13 months after second CR; 0–4 months of tislelizumab-containing combination therapy	► Second local relapse ► Third CR	► ICE chemotherapy ► Tislelizumab, dinutuximab beta, GM-CSF, and sequential mICE/VIT chemotherapy
Jan 2026 onward	After completion of four cycles of combination therapy; last reported follow-up in April 2026	► Persistent third CR	► Oral eflornithine maintenance therapy

Allo-HSCT, allogeneic hematopoietic stem cell transplantation; CR, complete response; ICE, ifosfamide, carboplatin, etoposide; INRGSS, international neuroblastoma risk group staging system; IT, irinotecan, temozolomide; NB, neuroblastoma; mICE, modified ifosfamide-carboplatin-etoposide; PD, progressive disease; PR, partial response; SCMC-NB-2016: A 2016 risk-stratified neuroblastoma protocol from Shanghai Children’s Medical Center. For MYCN-amplified high-risk patients, six-agent induction chemotherapy is given in alternating 21-day sequential cycles (maximum 10 cycles): cyclophosphamide plus topotecan (CTX+TOPO), CTX+TOPO, cisplatin plus etoposide (CDDP+VP16), cyclophosphamide plus doxorubicin plus vincristine (CTX+Doxo+VCR), CDDP+VP16, CTX+Doxo+VCR, CTX+TOPO, CDDP+VP16, CTX+Doxo+VCR, CTX+TOPO; SD, stable disease; VIT, vincristine, irinotecan, temozolomide.

Given the patient’s preserved performance status, tislelizumab-containing chemoimmunotherapy was initiated. The patient received four cycles of tislelizumab-containing combination chemoimmunotherapy from August 2025 to January 2026, commencing 3 months after confirmation of PD. During cycles 1 and 2, the patient received tislelizumab (3 mg/kg IV every 4 weeks), naxitamab (2.25 mg/kg IV on days 2, 4, 8 and 10), and modified ICE (mICE) chemotherapy. The mICE regimen consisted of ifosfamide 1,500 mg/m^2^/day IV on days 1–3, etoposide 100 mg/m^2^/day IV on days 1–3, carboplatin 400 mg/m^2^/day IV on day 1, and GM-CSF 250 μg/m^2^/day SC on days 4–10. Cycles 3 and 4 adopted tislelizumab plus naxitamab at identical dosing schedules, combined with the vincristine, irinotecan, and temozolomide (VIT) regimen, comprising vincristine 1.5 mg/m^2^ IV on day 1, irinotecan 50 mg/m^2^/day IV on days 1–5 concurrently with oral temozolomide 100 mg/m^2^/day and GM-CSF 250 μg/m^2^/day SC on days 6–12. Treatment was administered in 4-week cycles. After two cycles of the combination therapy, the product of three orthogonal diameters of the target lesion decreased by 66.9% (Figure 1C). After completion of four cycles of treatment, the local lesion was further reduced in size (Figure 1D). The bone marrow evaluation revealed CR on both cytology and flow cytometry. No blood product transfusions were required between August 2025 and January 2026. The patient maintained a good performance status during treatment.

### Patient 2

Patient 2 was diagnosed in May 2020, at 9 years of age, with MYCN non-amplified NB originating from the retroperitoneum. The disease was classified as stage M according to the INRG staging system. From May 2020 to June 2022, the patient completed intensive multimodal therapy, including 14 cycles of chemotherapy, surgery, radiotherapy, allogeneic hematopoietic stem cell transplantation (allo-HSCT), and three cycles of dinutuximab beta plus isotretinoin. She subsequently achieved first CR. Between August 2023 and September 2024, the patient experienced her first relapse, presenting with a newly detected soft-tissue lesion in the right frontal region. The patient received salvage treatment, including two surgical resections, 10 cycles of chemotherapy, two separate courses of local radiotherapy delivered before DFMO maintenance and prior to second disease progression, and 3 cycles of dinutuximab beta-based immunotherapy, after which she achieved a second CR. The first radiotherapy course was stereotactic body radiotherapy (SBRT) targeting the postoperative intracranial tumor bed in September 2023, and the second radiotherapy course utilized volumetric modulated arc therapy (VMAT) for postoperative mediastinal and pleural metastatic lesions in January 2024. Oral eflornithine (DFMO) maintenance therapy was administered from December 2024 to May 2025, and DFMO was discontinued in May 2025 before the second relapse and before initiation of the tislelizumab-containing regimen. After completion of the combination regimen, DFMO maintenance was resumed/continued according to the institutional maintenance strategy (Table 1).

**FIGURE 2:**
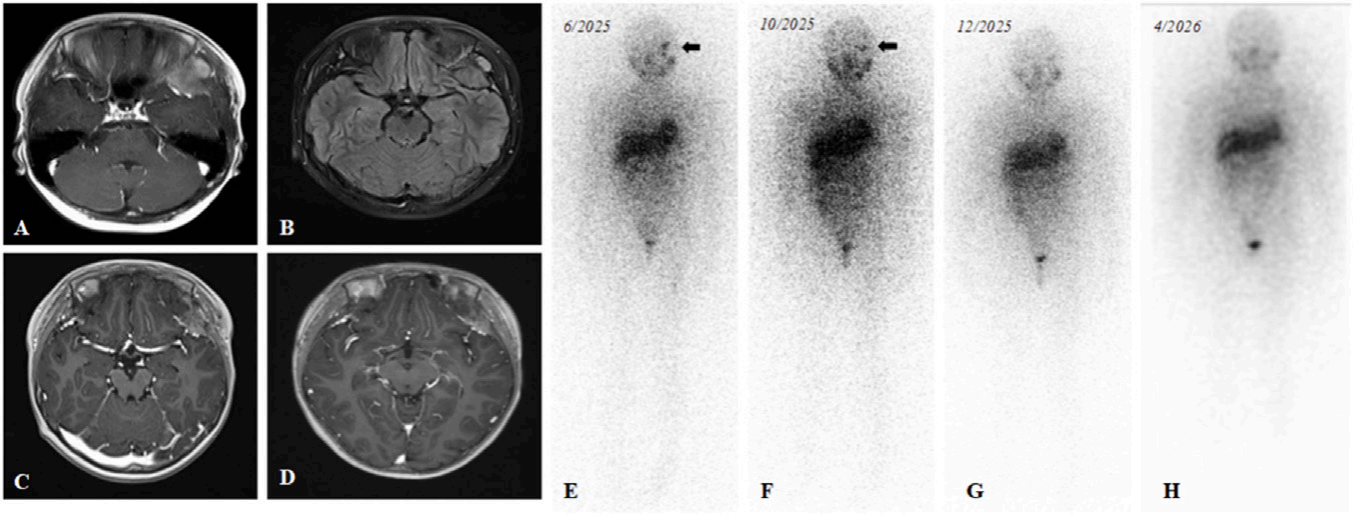
Imaging findings of Patient 2. Black arrows indicate the dominant soft-tissue lesion in the left temporal region. **(A)** MRI at the time of second relapse in July 2025; the local soft-tissue lesion measured 27.7 mm × 23.0 mm × 15.6 mm. **(B)** MRI after salvage ICE chemotherapy in August 2025. **(C)** MRI after two cycles of combination therapy in October 2025; the local soft-tissue lesion measured 24.4 mm × 17.6 mm × 8.2 mm. **(D)** MRI after four cycles of combination therapy in December 2025. **(E)** MIBG imaging at second relapse in June 2025. **(F)** MIBG imaging after two cycles of combination therapy in October 2025. **(G)** MIBG imaging in December 2025. **(H)** MIBG imaging in April 2026. No residual abnormal MIBG uptake was detected on images G and H, with complete regression of the previous metastatic lesion.

In June 2025, 13 months after entering second CR, the patient developed a second relapse manifesting as a left temporal extracerebral mass (Figure 2E). Two cycles of salvage ICE chemotherapy were initiated in July 2025, yet no radiographic tumor regression was observed 1 month after salvage chemotherapy initiation (Figures 2A,B). Given the lack of response to salvage ICE chemotherapy, tislelizumab-containing chemoimmunotherapy was administered from August to December 2025. The regimen was similar to that used for Patient 1, except that dinutuximab beta (10 mg/m^2^ IV on days 1–10, total dose 100 mg/m^2^, every 4 weeks) was used as the GD2-targeted antibody. After two cycles, the estimated lesion volume decreased by 64.5% (Figure 2C), and the MIBG Curie score decreased to 2 (Figure 2F). After four cycles of combination therapy, imaging in December 2025 demonstrated complete tumor resolution (Figure 2D). MIBG Curie score was 0 at this time point (Figure 2G) and remained 0 at 4 months after completion of the combination regimen (April 2026) (Figure 2H). The patient continued oral DFMO maintenance therapy and required no blood product transfusions between August 2025 and April 2026.

### Safety assessments

Treatment-emergent adverse events (TEAEs) were recorded during treatment and retrospectively graded according to CTCAE v5.0. Key safety endpoints, including infusion-related reactions, anti-GD2 antibody-associated pain, peripheral neuropathy, hematologic toxicities, hepatic and renal dysfunction, and thyroid abnormalities, are summarized in Table 2. Both patients received uniform supportive care, with corticosteroid prophylaxis strategies tailored to the specific anti-GD2 antibody administered.

**TABLE 2:** CTCAE v5.0-graded treatment-emergent adverse events during tislelizumab-containing anti-GD2 antibody-based chemoimmunotherapy.

Adverse events	Patient 1				Patient 2			
	Cycle 1	Cycle 2	Cycle 3	Cycle 4	Cycle 1	Cycle 2	Cycle 3	Cycle 4
Infusion-related reaction	0	0	0	0	0	0	0	0
Anti-GD2 antibody-associated pain	2	1	1	1	3	1	1	1
Peripheral neuropathy	0	0	0	0	0	0	0	0
Anemia	3	2	2	2	1	0	1	1
Neutrophil count decreased	0	2	1	1	3	1	1	1
White blood cell count decreased	1	1	1	1	2	0	0	1
Platelet count decreased	0	0	0	0	0	0	0	0
Hepatic enzyme elevation (ALT/AST)	0	0	0	0	0	0	0	0
Renal function impairment	0	0	0	0	0	0	0	0
Thyroid dysfunction (hypo-/hyperthyroidism)	0	0	0	0	0	0	0	0
Pyrexia	1	0	0	0	2	2	1	0
Gastrointestinal toxicity	1	0	0	0	2	1	1	0

CTCAE, common terminology criteria for adverse events; IRR, infusion-related reaction; mICE, modified ifosfamide-carboplatin-etoposide; VIT, vincristine, irinotecan, temozolomide. Cycles 1–2: mICE, backbone; Cycles 3–4: VIT, backbone. Patient 1: naxitamab + tislelizumab; Patient 2: dinutuximab beta + tislelizumab. Grade 0 = no adverse event observed.

Standardized supportive care was uniformly implemented in both patients to alleviate treatment-related toxicities. Preemptive oral gabapentin combined with ibuprofen oral solution was used to control GD2 antibody-associated pain, with intravenous sufentanil available as rescue analgesia and titrated downward per serial pain evaluations. Systemic antihistamines were given before each anti-GD2 antibody infusion to prevent hypersensitivity and infusion-related reactions (IRRs). Alongside mICE and VIT chemotherapy, routine antiemetics, gastric mucosal protectants, and individualized intravenous fluid support were administered to reduce chemotherapy-induced gastrointestinal adverse effects and preserve fluid-electrolyte balance. Laboratory assessments including complete blood counts, liver and renal biochemistry, and thyroid function tests were performed before every cycle for continuous toxicity surveillance. Corticosteroid administration followed protocol-defined distinctions between the two anti-GD2 regimens. For Patient 1 receiving naxitamab plus tislelizumab, only ultra-low-dose corticosteroids were allowed for preemptive prevention of potential IRRs during antibody infusion, and systemic steroids were not used to manage pain, myelosuppression, or other treatment-emergent adverse events (TEAEs). Per institutional guidelines, corticosteroids were entirely withheld from all prophylactic and therapeutic interventions throughout all four cycles of dinutuximab beta-based therapy in Patient 2. No grade ≥3 immune-related adverse events affecting endocrine, hepatic, neurologic, or cutaneous systems were detected in either patient. Neither participant experienced grade 2 or higher IRRs, peripheral neuropathy, sustained hepatorenal damage, or thyroid dysfunction. Myelosuppression and antibody-mediated pain were the most common TEAEs; all were transient and responsive to supportive measures, with no permanent treatment interruptions or discontinuation. Neither patient required red blood cell or platelet transfusions throughout the entire treatment course.

## Discussion

High-risk NB remains a major challenge in pediatric oncology because of its high relapse rate and limited response to salvage therapy (Moreno et al., 2025). Anti-GD2 antibodies constitute a cornerstone of frontline therapy and are also used in the immunotherapeutic management of R/R NB, and have improved clinical outcomes (Hernádfői et al., 2025; Ladenstein et al., 2020). The HR-NBL1 trial demonstrated that dinutuximab beta significantly improved survival outcomes; compared with standard maintenance therapy using isotretinoin alone, the 5-year overall survival (OS) increased from 50% to 64%, and the 5-year EFS rose from 42% to 57% (Ladenstein et al., 2020). Another study of naxitamab reported a 5-year EFS of 58% and a 5-year OS of 78.6% among high-risk NB patients who achieved first CR (Mora et al., 2023). Chemoimmunotherapy combining anti-GD2 antibodies with conventional chemotherapy has shown clinical activity in R/R NB. Specifically, the combination of dinutuximab with temozolomide and topotecan (the TTo regimen) yielded an objective response rate (ORR) of 30.2% in R/R NB (Gray et al., 2026). Additionally, the early administration of the HITS regimen (naxitamab plus GM-CSF, irinotecan, and temozolomide) achieved an ORR of 32% and a CR rate of 29% in similar patient populations (Muñoz et al., 2023). In a large retrospective cohort study of 146 patients with relapsed HRNB, the standard salvage regimen I/T/DIN/GM-CSF yielded an ORR of 49% (CR 29%) and a median progression-free survival (PFS) of 13.1 months; notably, responses deepened with continued therapy, with 41% of initial partial response and 33% of minor response patients ultimately achieving CR (Lerman et al., 2023).

Despite these advances, some patients still experience disease progression after anti-GD2 antibody-based combination therapy and continue to have poor outcomes. In the present study, Patient 1 received dinutuximab beta combined with chemotherapy upon disease recurrence. Patient 2 underwent frontline maintenance therapy with dinutuximab beta and isotretinoin, followed by dinutuximab beta at the first relapse; nevertheless, both patients experienced subsequent disease progression. In the present report, both patients achieved short-term clinical responses after initiation of the full multi-agent regimen consisting of tislelizumab, anti-GD2 antibody, GM-CSF support, and chemotherapy. However, given the absence of a control group receiving anti-GD2-based chemoimmunotherapy without tislelizumab, these observations cannot establish whether the PD-1 inhibitor contributed incremental efficacy beyond that attributable to the chemoimmunotherapy backbone alone. The responses observed may reflect the activity of the anti-GD2 antibody-based chemotherapy regimen, the added effect of PD-1 blockade, or a combination thereof; this uncertainty is inherent to the case-report design and underscores the need for prospective controlled studies to isolate the specific contribution of PD-1 inhibition in this setting. Together with published preclinical and clinical evidence, our observations are consistent with the rationale for further evaluating immune checkpoint inhibitors as part of combination strategies in R/R high-risk NB (Topalian et al., 2015) and warrant formal evaluation in clinical trials.

PD-1 is an immune checkpoint receptor predominantly expressed on activated T and natural killer (NK) cells, whose ligation with PD-L1/PD-L2 blunts endogenous antitumor immune surveillance (Topalian et al., 2015; Van Dam et al., 2015). Neuroblastoma typically exhibits low constitutive PD-L1 expression, though PD-L1 upregulation can be induced by antibody-dependent cellular cytotoxicity (ADCC) triggered by anti-GD2 antibody binding to NB cell membranes (Boes and Meyer-Wentrup, 2015; Dondero et al., 2016). Preclinical studies suggest potential synergistic immune activation when PD-1 blockade is combined with GD2-targeted immunotherapy: anti-GD2 antibodies drive robust NK cell-mediated tumor killing while simultaneously upregulating PD-1 on effector lymphocytes and PD-L1 on residual tumor cells, creating a targetable immune checkpoint axis (Siebert et al., 2017; Siebert et al., 2023). These mechanistic laboratory findings provide a strong biological rationale for the triple chemoimmunotherapy strategy evaluated herein; however, preclinical synergy does not reliably translate to measurable incremental clinical efficacy in pediatric solid tumors, and real-world clinical evidence supporting added value of PD-1 inhibitors to anti-GD2 chemoimmunotherapy remains extremely scarce. Multiple phase I/II trials have confirmed limited single-agent activity of PD-1 blockade in unselected pediatric solid tumors, and PD-L1 biomarker status alone fails to reliably identify patients who may derive clinical benefit from checkpoint inhibition (Geoerger et al., 2020). Recent reviews of pediatric NB immunotherapy also highlight the poor single-agent efficacy of PD-1 inhibitors and underscore the necessity of dual immunotherapy combining GD2 targeting and checkpoint blockade to reverse local immune suppression (Mao et al., 2024). The two anecdotal case observations presented here merely serve as preliminary hypothesis-generating signals and cannot be interpreted as proof of synergistic clinical activity for the tislelizumab-containing triple regimen.

Given the immune-activating properties of both anti-GD2 antibodies and PD-1 inhibitors, immune-related adverse events remain a potential concern. Therefore, close monitoring for immune-related toxicities is necessary. Tislelizumab is a humanized anti-PD-1 monoclonal antibody with a manageable safety profile in adult malignancies; however, pediatric experience remains limited. No grade ≥3 immune-related organ toxicity was observed. Grade 3 hematologic toxicity and anti-GD2 antibody-associated pain occurred but were transient and manageable with supportive care, suggesting that this combination may have an acceptable safety profile in selected pediatric patients.

Several limitations of this study should be acknowledged. First, both patients received a complex multi-agent regimen incorporating tislelizumab, an anti-GD2 monoclonal antibody, GM-CSF support, and chemotherapy. No separate control cohort receiving single-agent tislelizumab or anti-GD2 chemoimmunotherapy without PD-1 blockade was included. Consequently, we cannot isolate and quantify the independent antitumor efficacy exclusively attributed to tislelizumab, and the observed tumor regression cannot be attributed to PD-1 inhibition alone. The clinical activity we report only reflects the overall effect of the full combined regimen rather than the standalone benefit of tislelizumab. Second, this report included only two patients, which precludes definitive conclusions regarding the optimal dosing, treatment duration, efficacy, or safety of this regimen. Third, the follow-up period was short, and longer observation is needed to assess durability of response, late relapse, and delayed toxicities. Fourth, only Patient 1 received one-time PD-L1 testing at biopsy (TPS <1%, CPS = 5). Serial immune profiling including dynamic PD-L1 levels, tumor immune infiltrates, NK activity, lymphocyte subsets and cytokines was missing for both patients, limiting mechanistic interpretation of treatment responses. Future trials should incorporate longitudinal immune monitoring to identify patients who may be more likely to benefit from this combination strategy.

## Conclusion

We report two heavily pretreated pediatric patients with R/R high-risk NB who received tislelizumab-containing anti-GD2 antibody-based chemoimmunotherapy. Both patients showed short-term clinical responses during the reported follow-up period, and treatment-related toxicities were manageable. However, because multiple active agents were administered simultaneously and no control group was included, the independent contribution of tislelizumab cannot be determined. These observations should be interpreted as preliminary, hypothesis-generating evidence supporting further prospective evaluation of this combination approach.

## Data Availability

The original contributions presented in the study are included in the article/supplementary material, further inquiries can be directed to the corresponding author.
